# Cross-cultural adaptation and validation of the Kerlan-Jobe Orthopaedic Clinic shoulder and elbow score in Finnish-speaking overhead athletes

**DOI:** 10.1186/s13102-022-00581-4

**Published:** 2022-11-07

**Authors:** Maria Sukanen, Jesse Pajari, Sami Äyrämö, Juha Paloneva, Benjamin Waller, Arja Häkkinen, Juhani Multanen

**Affiliations:** 1 Faculty of Sport and Health Sciences, University of Jyväskylä, P.O. BOX 35, 40014 Jyväskylä, Finland; 2 The Finnish Sports Physiotherapists Association, Helsinki, Finland; 3 Faculty of Information Technology, University of Jyväskylä, Jyväskylä, Finland; 4 Department of Surgery, Central Finland Health Care District, Jyväskylä, Finland; 5 University of Eastern Finland, Kuopio, Finland; 6 Physical Activity, Physical Education, Sport and Health Research Centre (PAPESH), Sport Science Department, School of Science and Engineering, Reykjavik University, Reykjavik, Iceland; 7 Department of Physical Medicine and Rehabilitation, Central Finland Health Care District, Jyväskylä, Finland

**Keywords:** Kerlan-Jobe, Shoulder, Overhead athlete, Cross-cultural adaptation, Validation, Finnish language

## Abstract

**Background:**

The Kerlan-Jobe Orthopaedic Clinic Shoulder and Elbow score (KJOC) is developed to evaluate the shoulder and elbow function in overhead athletes. To date, the score has not been adapted into Finnish language. The aim of this study was to perform a cross-cultural adaptation of the Kerlan-Jobe Orthopaedic Clinic Shoulder and Elbow score (KJOC) into Finnish language and evaluate its validity, reliability, and responsiveness in overhead athletes.

**Methods:**

Forward–backward translation method was followed in the cross-cultural adaptation process. Subsequently, 114 overhead athletes (52 males, 62 females, mean age 18.1 ± 2.8 years) completed the Finnish version of KJOC score, Disabilities of the Arm, Shoulder and Hand (DASH), American Shoulder and Elbow Surgeons Standardized Shoulder Assessment Form (ASES) and RAND-36 to assess validity of the KJOC score. To evaluate reliability and responsiveness, the participants filled in the KJOC score 16 days and eight months after the first data collection. Validity, reliability, and responsiveness of the Finnish KJOC score were statistically tested.

**Results:**

Minor modifications were made during the cross-cultural translation and adaptation process, which were related to culture specific terminology in sports and agreed by an expert committee. Construct validity of the KJOC score was moderate to high, based on the correlations with DASH (*r* = − 0.757); DASH sports module (*r* = − 0.667); ASES (*r* = 0.559); and RAND-36 (*r* = 0.397) questionnaires. Finnish KJOC score showed excellent internal consistency (*α* = 0.92) and good test–retest reliability (2-way mixed-effects model ICC = 0.77) with acceptable measurement error level (SEM 5.5; MDC 15.1). Ceiling effect was detected for asymptomatic athletes in each item (23.2–61.1%), and for symptomatic athletes in item 5 (47.4%). Responsiveness of the Finnish KJOC score could not be confirmed due to conflicting follow-up results.

**Conclusion:**

The Finnish KJOC score was found to be a valid and reliable questionnaire measuring the self-reported upper arm status in Finnish-speaking overhead athletes.

**Supplementary Information:**

The online version contains supplementary material available at 10.1186/s13102-022-00581-4.

## Background

Shoulder and elbow pain and injuries originating from athletic overuse are well described in sports such as baseball [1], swimming [2], and volleyball [3]. Physical demands directed to these joints in overhead sports may lead to chronic loading, affecting athletic performance [4, 5]. The consequential symptoms might be detectable only during sport-specific training or competing, without deficiencies in daily life activities [6]. Athletes appear to continue to train and compete despite upper arm symptoms [7, 8]. A diversity in shoulder and elbow symptom perceptions creates a challenge both for athletes and healthcare professionals to evaluate and monitor the upper extremity health and performance.

Subjective evaluation of patient’s experiences is used to complement clinical outcomes or serve as a primary measure when objective results cannot be obtained [9, 10]. Several patient-rated outcome measures for upper arm health have originally been developed for the general population [11]. These tools have commonly been used for the athlete assessment [12, 13]. However, questionnaires developed for general use might not be specific enough to detect minor and slowly evolving changes in the athletic performance. This might lead to underestimating the athlete’s functional deficiencies and therefore make them more vulnerable to subsequent injury [6, 12, 14].

The Kerlan Jobe Orthopaedic Clinic (KJOC) Shoulder and Elbow score is a sport-specific questionnaire developed to assess the upper arm health of overhead athletes. KJOC score enables sensitive observation of subtle changes in athletes’ shoulder and elbow function and performance [6]. Detection of functional changes may aid in planning the sports training, rehabilitation, and return to sports after injury. The KJOC score is a valid and reliable tool in English speaking overhead athletes [6, 15, 16], and it has recently been validated to several other languages [17–22]. However, the validity, reliability, and responsiveness of the Finnish version of KJOC have not been previously reported. This study aimed to produce a Finnish version of the KJOC score and evaluate its psychometric properties. We hypothesized that the KJOC score would be a valid, reliable, and responsive tool to assess the Finnish-speaking overhead athlete’s shoulder and elbow functionality.

## Methods

### Translation and cross-cultural adaptation

Before conducting the research, the developer of the original KJOC score was contacted to obtain permission to use the English language questionnaire. Cross-cultural adaptation was performed following published guidelines [23]. Two independent translators, an informed and an uninformed professional, performed the translation from English to Finnish. The translations were united, and the resulting synthesis was discussed by a research committee formed of specialists from health sciences, orthopaedics, physical therapy, and overhead sports coaching. The back-translation was executed by a native English speaker fluent in the Finnish language, who was not familiar with the original questionnaire. Subsequently, the modified translation was piloted with ten overhead athletes and revised due to gathered observations. Progression of the cross-cultural adaptation process was documented, and its’ description can be provided upon a request.

### Study population and recruitment

Study population was recruited through sports clubs from the Capital region of Finland and the Jyväskylä region in Central Finland. The inclusion criteria were age over 15 years, currently active status in overhead sports, and Finnish as first language. The exclusion criterion was a recent upper limb or another injury that prevented active participation in sports. A total of 118 athletes were recruited, and 114 found eligible for the study. Four athletes were excluded due to missing values, missing written consent, a neurological condition that could have influenced the data of interest, and under aged person [14 years old]. Included participants were athletes from five different overhead sports (volleyball, swimming, tennis, gymnastics, Finnish baseball) competing in national or international level. The research was undertaken with ethical approval from the Human Sciences Ethics Committee of the University of Jyväskylä (147/13.00.04.00/2020). Participants provided written consents prior to participation and rights of the participants were protected throughout the study.

### Data collection

Questionnaires were administered on three occasions during a time-period when all athletes were in active training and competing (Table 1). Between sport specialisations, pre-season and competition seasons were scheduled somewhat differently during the study. To evaluate the validity of the Finnish KJOC score, baseline measurements were performed during September 2020 after the first wave of Covid-19 pandemic had stabilised in Finland. Athletes were asked to fill in printed versions of KJOC, Disabilities of the Arm, Shoulder and Hand (DASH), American Shoulder and Elbow Surgeons Standardized Shoulder Assessment Form (ASES), and RAND-36 questionnaires [24–27] to assess construct validity. Additionally, FIT (frequency, intensity, time) index of Kasari, and questions related to Covid-19 pandemic were inquired. KJOC score was distributed again after two weeks to test the test–retest reliability of the survey, alongside a question inquiring possible alterations in physical health within the two time points. Responsiveness of the KJOC survey was implemented eight months after baseline. Questionnaires were mainly returned by mail, with the remaining being collected during athletes’ training.

**Table 1:** Data collection with used questionnaires at each timepoint

Questionnaire	Baseline	Re-test	Responsiveness
KJOC	x	x	x
ASES	x		
DASH	x		
RAND-36	x		
Effect of Covid-19	x		x
FIT Index of Kasari	x		

### Questionnaires

The ten-item KJOC score is formed of two sections, both including five questions that inquire athlete’s perceptions about their shoulder and elbow function and performance. Respondents place the answer to each item with a mark on a ten-centimeter Visual Analog Scale (VAS). Left margin of the scale stands for zero points and the right side for ten points, a higher score indicating better function of the upper arm. Item scores are measured with a ruler starting from the left corner of the scale to the mark placed by the participant and recorded in centimetres with one decimal. Overall score is the sum of all items, resulting in a score between 0 and 100 points with 100 points standing for perfect upper extremity function [6].

ASES, DASH and RAND-36 were used as reference questionnaires to assess the construct validity of the Finnish KJOC score. ASES is used to assess daily shoulder disability and pain with overall score ranging between 0 and 100 with higher result resembling better functionality [28]. DASH questionnaire evaluates function of the entire upper arm, and is scored between 0 and 100, with lower score indicating better upper arm health. Optional sport module (DASH-SM) comprising four questions related to free-time activities addresses upper arm function more comprehensively in physically active individuals [29]. RAND-36 score evaluates different sectors of general quality of life with eight subscales: (1) physical functioning, (2) role limitations due to physical problems, (3) role limitations due to emotional problems, (4) energy/fatigue, (5) emotional well-being, (6) social functioning, (7) pain, and (8) general health. Overall score is calculated within 0–100 points, with higher result describing better quality of life [30]. All reference scores have been previously validated into Finnish.

### Descriptive data

Alongside KJOC-score and reference questionnaires, participants were asked to fill in questions regarding general physical activity level with the FIT Index of Kasari [31] (scale between 0 and 100, higher score describing higher physical activity). In addition, due to the outbreak of Covid-19 pandemic during spring 2020, the influence of pandemic on sports training was inquired with following questions: How the pandemic affected the amount of (1) leisure-time physical activities; (2) Exercise within your sporting event; and (3) What was the trend of adjustments in the training? The Covid-19 questions were repeatedly inquired also at the responsiveness timepoint.

### Statistical methods

Construct validity of the KJOC score was tested to evaluate the ability of the instrument to measure the phenomenon it was created to measure [32]. Correlations between the KJOC score and reference questionnaires were determined with Pearsons correlation coefficients and their corresponding p-values [33]. Moderate to strong correlations were hypothesized to be detected between the KJOC and other upper arm questionnaires. In contrast, correlation with RAND-36 was expected to be weak to moderate, since the instrument measures a divergent construct. Further, construct validity was also assessed by measuring how the KJOC score discriminates respondents according to their self-reported subgroup of upper arm function: 1. playing without any arm trouble, 2. playing, but with arm trouble, or 3. not playing due to arm trouble. Independent samples *t*-test was used in investigating the statistical significance of mean score differences between subgroups.

Reliability was determined for single items and overall KJOC score by the test–retest procedure [32]. Intraclass correlation coefficient (ICC) with corresponding 95% confidence intervals (CI) [34] were computed based on absolute-agreement, using a 2-way mixed effects average measures model. Measurement error was determined with the standard error of measurement (SEM) with formula *SEM* = *standard deviation (SD)* × *(√1 − ICC)*[35]. Subsequently, minimal detectable change (MDC) was calculated with *MDC* = *SEM* × *1.96* × *√2* Eq. [36]. Bland–Altman plot with 95% limits of agreement was used to plot the mean difference between the test–retest scores against the mean of the two measurements [37]. Internal consistency of the KJOC score was determined to assess mutual uniformity of different sections of the score [32] by calculating values of Cronbach’s alpha coefficient [36].

In responsiveness analysis, it was determined if KJOC score detects physiological changes in upper extremity function after time [38]. Score difference was evaluated for those respondents who reported a change in their upper arm health between baseline and responsiveness measurements. The change in upper arm health was inquired with Global Rating of Change scale (GRC). Wilcoxon signed-rank test was used to detect statistical significance between the KJOC total mean scores. Furthermore, standardized response mean (SRM) and effect size (ES) were computed. SRM was evaluated by calculating the difference between the baseline and follow-up mean scores and dividing the difference by SD of the difference. ES was determined by calculating the difference between the follow-up and baseline mean scores, divided by the baseline measurement SD [38, 39].

### Floor- and ceiling effect

Floor- and ceiling effects were detected as evident if more than 15% of the study subjects scored either highest or lowest possible score within one item. If more than 25% of the score items showed a floor or ceiling effect, the whole score was concluded to present the phenomenon [40].

All statistical analyses were performed with IBM SPSS Statistics 24.0 for Windows software. Descriptive statistics were calculated and reported for all relevant measures and presented as means and standard deviations for continuous variables and as counts and percentages for other variables. The normality of variables was evaluated graphically and with the Shapiro–Wilk W test.

## Results

### Cross-cultural adaptation

Cross-cultural adaptation revealed minor cultural differences. The options to describe athletes’ level of competition (*professional major league, professional minor league, intercollegiate, high school*) were adopted to match Finnish sporting culture classifications with terms that may be translated as *professional-, semi-professional- and recreational athlete.* Further, words *game* and *playing* were translated into Finnish to match the words *competition* and *competing.* The latter translations were chosen since the terms acknowledge both team- and individual performance sports, and they were not considered altering the primary concept of the items in question. From the KJOC item five, the expression *traded or waived* was eliminated since trading or waiving the athletes does not occur in Finnish sporting culture. The questionnaire was considered straightforward and easy to use among the pilot test population. The back-translation of the Finnish KJOC questionnaire is available in an additional file (see Additional file:  1).

### Study participants

Demographic and clinical characteristics of the participants are presented in Table 2.

**Table 2:** Athlete characteristics

Demographic	Baseline *n* = 114	Re-test *n* = 76	Responsiveness *n* = 38
Age, mean (SD)	18.1 (2.8)	18.3 (3.0)	18.2 (3.1)
Females, *n* (%)	62 (54.4)	47 (61.8)	16 (42.1)
BMI, mean (SD)	22.5 (2.1)	–	–
Level of education			
Preliminary school, n (%)	17 (14.9)	–	–
High school/occupational education	74 (64.9)	–	–
University	23 (20.2)	–	–
Dominant side, *n* (%)			
Right	101 (88.6)	68 (89.5)	35 (92.1)
Left	10 (8.8)	6 (7.9)	3 (7.9)
Ambidextrous	2 (1.8)	2 (2.6)	–
Sport, *n* (%)			
Swimming	58 (50.9)	32 (42.1)	23 (60.5)
Volleyball	36 (31.6)	32 (42.1)	10 (26.3)
Artistic gymnastics	9 (7.9)	5 (6.6)	0
Tennis	8 (7.0)	5 (6.6)	5 (13.2)
Finnish baseball	3 (2.6)	2 (2.6)	0
Level of sport, *n* (%)			
Professional	20 (17.5)	12 (15.8)	14 (36.8)
Semi-professional	91 (79.8)	61 (80.3)	23 (60.5)
Recreational	2 (1.8)	2 (2.6)	1 (2.6)
Years competing, mean (SD)	9.1 (2.8)	9.2 (3.0)	10.6 (3.5)
Arm currently injured, *n* (%)			
Yes	5 (4.4)	5 (6.6)	4 (10.5)
No	109 (95.6)	71 (93.4)	34 (89.5)
Upper arm status in sport, *n* (%)			
Playing with arm trouble	19 (16.7)	13 (17.1)	7 (18.4)
Playing without arm trouble	95 (83.3)	63 (82.9)	30 (78.9)
Not playing due to arm trouble	0	0	1 (2.6)
Missed time in competition/practice in the last year due to an arm injury, *n* (%)			
Yes	27 (23.7)	17 (22.4)	10 (26.3)
No	87 (76.3)	59 (77.6)	28 (73.7)
Received treatment due to an arm injury, *n* (%)			
Yes	41 (36.0)	29 (25.4)	15 (39.5)
No	61 (53.5)	45 (39.5)	20 (52.6)
Level of physical activity (Kasari FIT Index)			
Exercising > 6 × week, > 30 min at a time	102 (89.5)	–	–
Intensity of exercising high/vigorous	93 (81.6)	–	–
MET h/week > 16	114 (100)	–	–
MET h/week > 33	87 (76.3)	–	–
Influence of Covid-19 on sports training, *n* (%)			
No influence	75 (65.8)	–	14 (36.8)
Training decreased	7 (6.2)	–	22 (57.9)
Training increased	22 (19.3)	–	1 (2.6)
Trend of Covid-19 influence on sports training, *n* (%)			
Less team training, more independent workouts	46 (40.4)	–	22 (57.9)
All training decreased	5 (4.4)	–	3 (7.9)
All training increased	11 (9.6)	–	1 (2.6)

*N* Number of participants, *SD* Standard deviation, *BMI* Body mass index, *MET* Metabolic equivalent of task

### Validity

The Finnish KJOC score showed a high correlation with DASH and moderate correlations with DASH-SM, ASES, and RAND-36 scores (Table 3). Furthermore, RAND-36 subscale correlations were computed as negligible to low (*r* = 0.105–0.457), with one subscale correlation resulting in statistically insignificant (role limitations due to emotional problems, *p* = 0.266). The subgroup analysis indicate that all correlations were higher for symptomatic athletes than asymptomatic (Table 3).

**Table 3:** ASES, DASH, and RAND-36 score results, and pearsons correlation coefficients with the finnish KJOC score

		ASES	DASH	DASH-SM	RAND-36
All participants	*n*	108	114	90	112
	*r*	0.559**	− 0.757**	− 0.667**	0.397**
	mean (SD)	95.5 (7.1)	3.4 (5.1)	5.9 (14.2)	82.5 (13.1)
Asymptomatic	*n*	91	95	75	93
	*r*	0.353**	− 0.502**	− 0.304**	0.314**
	mean (SD)	96.7 (6.1)	2.4 (2.9)	3.4 (9.7)	83.5 (12.7)
Symptomatic	*n*	17	19	15	19
	*r*	0.653*	− 0.773**	− 0.779**	0.584*
	mean (SD)	89.2 (8.6)	8.6 (9.1)	18.3 (24.3)	77.6 (14.1)

*n* Number of participants, *r* Pearsons correlation coefficient, *SD* Standard deviation, *p < 0.05; **p < 0.001

In cross-category comparisons, statistically significant differences in KJOC mean scores were detected between all subgroups. The mean scores of symptomatic athletes (72.4 ± 19.4; 95%CI 63.1–81.7) were significantly lower than those of asymptomatic athletes (92.6 ± 6.6; 95%CI 91.3–94.0; *p* < 0.001). Similarly, participants who had either lost time in training or competition within the past year because of arm trouble (83.1 ± 18.1 vs 91.2 ± 9.3; *p* = 0.003) or who had received care due to an upper arm injury (84.4 ± 15.7 vs 90.9 ± 9.5; *p* = 0.01), scored lower compared to their counterparts.

### Reliability

#### Internal consistency

The internal consistency regarding the ten items of the Finnish KJOC score was evaluated as excellent (*α* = 0.92), indicating a good homogeneity within the questionnaire.

#### Test–retest reliability

**Fig. 1:**
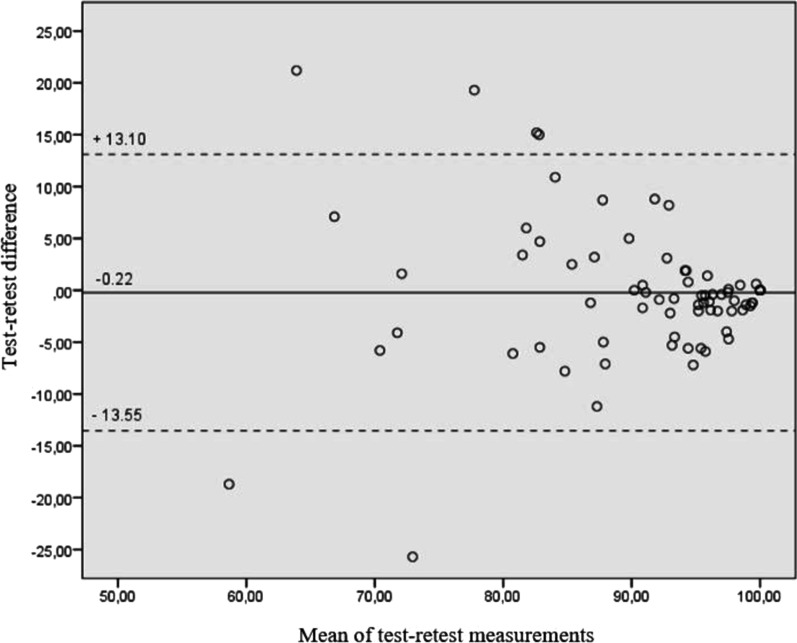
Bland–Altman’s plot describing the test–retest reliability

The test–retest data was collected after median time interval of 16 days. The ICC of the total KJOC score was 0.77 with values ranging between 0.38 and 0.77 for single items (*p* < 0.001) (Table 4). SEM and MDC were calculated as 5.5 and 15.1 for the whole study population. The Bland–Altman’s plot showed a small mean difference of −0.22, and 95% limits of agreement ranging from − 13.55 to 13.10 (Fig. 1).

**Table 4:** Test–retest reliability of the Finnish KJOC score for the total score, and single items

	Test score mean (SD)	Retest score mean (SD)	ICC (95% CI)
Total score	89.3 (12.4)	90.9 (10.1)	0.77 (0.66–0.85)
Item 1 (warming up)	8.1 (2.3)	8.4 (2.0)	0.38 (0.17–0.56)
Item 2 (pain)	8.5 (1.8)	8.7 (1.8)	0.77 (0.66–0.85)
Item 3 (weakness)	8.6 (1.8)	8.8 (1.6)	0.72 (0.59–0.81)
Item 4 (instability)	9.2 (1.5)	9.2 (1.2)	0.41 (0.21–0.58)
Item 5 (team relations)	9.7 (0.7)	9.8 (0.7)	0.51 (0.32–0.66)
Item 6 (technique)	8.8 (1.8)	9.1 (1.3)	0.67 (0.52–0.78)
Item 7 (speed/power)	9.2 (1.4)	9.3 (1.1)	0.58 (0.41–0.71)
Item 8 (endurance)	9.2 (1.5)	9.4 (1.1)	0.68 (0.54–0.79)
Item 9 (movement control)	8.9 (1.7)	9.1 (1.5)	0.55 (0.37–0.69)
Item 10 (competitive level)	9.1 (1.5)	9.2 (1.3)	0.67 (0.52–0.78)

*SD* standard deviation, *ICC* Intraclass correlation coefficient, *CI* Confidence interval

### Floor and ceiling effects

Within symptomatic athletes, the Finnish KJOC score showed a ceiling effect with item five (47.4%), which inquires the athletes’ relations with team coaches and management. Instead, a ceiling effect of the whole score was observed for asymptomatic athletes, with 23.2% to 61.1% giving the highest score for each item. Floor effect was not detected in symptomatic nor asymptomatic athletes.

### Responsiveness

The responsiveness data was collected after median time interval of eight months. Twenty-four out of 38 respondents reported a change in upper arm functional status with GRC scale either for better or for worse after the follow-up period. Changes in total KJOC scores resulted conflicting since a significant decline in mean scores was detected disregard the reported trend of change. Also, SRM and ES values supported the significance and quantity of the mean score changes. No significant differences were detected in participant characteristics (Table 5).

**Table 5:** Responsiveness of the finnish KJOC score

Subgroup	Baseline mean (SD)	Follow-up mean (SD)	Mean score change from baseline to follow-up (SD)	*p*-value	SRM	ES
Change for better (*n* = 15)	88.1 (13.0)	80.1 (8.3)	8.0 (11.4)	0.023	0.70	0.61
Change for worse (*n* = 9)	94.6 (3.1)	77.4 (14.8)	17.2 (13.2)	0.008	1.31	5.56
No change (*n* = 14)	96.2 (5.1)	96.9 (4.5)	−0.76 (3.1)	0.388	− 0.24	− 0.15

*SD* Standard deviation, *SRM* Standardized response mean, *ES* Effect size, *n* Number of participants

## Discussion

This study reports the cross-cultural adaptation and validation of the KJOC score into Finnish language. The questionnaire was shown to be a valid and reliable measure in evaluating the functionality and performance of the shoulder and elbow in Finnish-speaking overhead athletes.

The face- and content validity of the original KJOC-score were ensured throughout the standardized process of cross-cultural adaptation [23]. Few cultural and linguistic adjustments were necessary to produce a conceptually equivalent version of the original questionnaire. Athletes perceived the translation easy to understand and complete.

Detected correlations with DASH, DASH-SM and ASES corresponded the early hypothesis of moderate to strong correlations and the results were in line with previous studies [6, 17–22]. In subgroup analysis, correlations resulted higher within symptomatic subjects. Asymptomatic athletes might not show symptoms with general scores but instead report minor changes in upper arm performance with KJOC. Whereas, symptomatic subjects result with lower results in all upper arm questionnaires. Although ASES does not detect elbow impairments, it was selected to the study setting due to its´ previous use in athletes´ shoulder conditions [12, 13]. Hence, detected correlation between KJOC and ASES relates to KJOC score’s ability to detect shoulder symptoms. Previously one study [22] has reported the divergent validity of the KJOC, respectively. Similarly, with the previous results, a moderate correlation between the Finnish KJOC score and RAND-36 was detected, as hypothesized. Sport represents a considerable role in athletes’ daily life, and the moderate correlation may describe a link between perceptions regarding general health and physical performance.

Construct validity was supported by the KJOC score’s ability to stratify athletes by their self-reported upper arm functional status. The KJOC overall score showed wider mean score difference between symptomatic and asymptomatic athletes compared to the other upper arm scores. In addition, symptomatic athletes’ total KJOC scores varied more extensively from the highest score possible compared to asymptomatic athletes. These observations are parallel with the original idea behind the KJOC score to function as a sensitive tool between overhead athletes with or without upper arm insufficiencies [6]. Overall, the correlations and differentiating abilities suggest a good construct validity of the Finnish KJOC score.

Internal consistency was evaluated as excellent, and is in line with previous studies [17–21]. Compared to earlier publications, ICC resulted lower (0.77 vs. 0.82–0.99) and consequently, the measures of absolute reliability were of a higher level (SEM 5.5 vs. 0.81–8.54; MDC 15.1 vs. 2.42–8.5) [6, 17, 19–22]. The variation in repeatability results might be due to the characteristics of study subjects. In general, low mean age (18.1), high participation of asymptomatic athletes (83.3%), and athletes’ semi-professional level of competition (79.8%) might have resulted in the increased variability in the test–retest data. Previously, it has been argued that older and higher-level athletes might give more precise answers related to their athletic performance [20]. Older and higher-level athletes might possess a broader range of symptoms and observe themselves in a closer manner, leading to differences in the accuracy of reporting.

The Finnish KJOC score showed an apparent ceiling effect in asymptomatic subjects, consistent with two earlier translations [19, 21]. These findings can be considered expected when utilizing the KJOC score in primarily healthy athletes. Although, ceiling effect could also indicate a score’s weaker ability to identify mild functional changes. Within the symptomatic subjects, a ceiling effect was observed only in item five (47.4%), which inquired about the athletes’ relationship with club personnel. Hence, as the item does not measure shoulder or elbow functions, the symptomatic athletes may score generally high results within this item.

In addition to validity and reliability of the novel Finnish KJOC score, we evaluated responsiveness of the translation. Previously KJOC score’s responsiveness has been assessed in two studies evaluating the characteristics of the questionnaire before and after treatment with an average of 14- and 6-month time-intervals [6, 17]. In the present study setting, recruitment of injured athletes was not possible and the change in upper arm health status was determined by subjective rating in the eight-month follow-up time-point. Interestingly, not only athletes reporting their upper arm functional status as worse compared to baseline measurements, but also the ones rating their function better, resulted as significantly lower KJOC mean scores in follow-up. According to previous publications [6, 17], KJOC has shown to be a responsive measure after injury and a period of treatment, but remains unclear if longitudinal assessment of change in function is reliable in actively competing athletes with continuously experienced subtle symptoms. Detection of score change might be more reliable in cases where physical condition has experienced major changes between the data collection time-points. Before the Finnish KJOC score can be reliably used in the assessment of athlete recovery and long-term assessment of upper arm function and performance, the responsiveness of the score needs to be further studied.

There are some limitations of this study. The participants were recruited exclusively through sports club contacts, which led to the unequal distribution between asymptomatic (*n* = 95) and symptomatic (*n* = 19) athletes. Besides swimming and volleyball, the number of athletes from other overhead sports was also low. Further, since athletes were not asked about the location of upper arm symptoms, it remains unknown how many of the symptomatic subjects presented with shoulder or elbow insufficiency. Due to recruitment methods, it also cannot be concluded how the Finnish KJOC score would function with severely injured athletes in health care environment. Overall, the study was executed during the Covid-19 pandemic, and data was collected within the altering guidelines of national restrictions. Athletes reported changes in the quantity and quality of sports training, and it is justifiable to consider, if collected KJOC score results would have differed if measured during another time-period. Despite the unusual circumstances, results regarding the psychometric properties of the Finnish KJOC score are still to be considered reliable, since they describe the score properties instead of athlete function and performance. As a strength of this study, the included study population was considered sufficient according to literature recommendations [36] and previously published validation studies of the KJOC score.

## Conclusions

The Finnish version of the KJOC score was evaluated as a valid and reliable questionnaire to measure the self-reported functionality of the shoulder and elbow of overhead athletes. The findings of this study indicate that the Finnish KJOC score may function as a useful tool in the evaluation of overhead athletes’ upper arm performance to identify possible impairments. The score may be applied to the athlete evaluation in training environments. Further studies from different overhead sports with broader sample sizes are required to develop more comprehensive information regarding the validity and feasibility of the Finnish KJOC score. In addition, further studies regarding the responsiveness of the score are warranted.

## Supplementary Information

**Additional file 1.** Back-translation of the Finnish KJOC score.

## Data Availability

The datasets generated and analyzed during the current study are not publicly available due to privacy protection policies. Datasets as well as the documented protocol of the cross-cultural adaptation process are available from the corresponding author on reasonable request.

## Abbreviations

ASES: American shoulder and elbow surgeons standardized shoulder assessment form
CI: Confidence interval
DASH: Disabilities of the arm, shoulder and hand
ES: Effect size
GRC: Global rating of change scale
ICC: Intraclass correlation coefficient
KJOC: Kerlan Jobe orthopaedic clinic shoulder and elbow score
MDC: Minimal detectable change
RAND-36: RAND-36 questionnaire of quality of life
SEM: Standard error of measurement
SRM: Standardized response mean
