# Supplementary material for: Cross-cultural adaptation and validation of the Kerlan-Jobe Orthopaedic Clinic shoulder and elbow score in Finnish-speaking overhead athletes
Source: BMC Sports Sci Med Rehabil. 2022 Nov 7;14:190. doi: 10.1186/s13102-022-00581-4 (PMC9640805; doi:10.1186/s13102-022-00581-4)
Supplement: Supplementary file 1 — Additional file 1. Back-translation of the Finnish KJOC score. [file 13102_2022_581_MOESM1_ESM.pdf]

Kerlan-Jobe shoulder and elbow questionnaire

(Kerlan-Jobe Orthopaedic Clinic Shoulder & Elbow Score, KJOC)

Name ________________________ Age _____ Sex/Gender____ Date ____________

Dominant hand (R) ______ (L) ______ (Ambidextrous) _______________

Sporting Event _____________ Position___________ Active competitive years _______

Answer the following questions related to your upper limb. In this questionnaire upper limb refers to your shoulder and elbow regions

	YES	No
1. Do you currently have an injury in your upper limb?	☐	☐
2. Are you currently actively participating in your sport?	☐	☐
3. In the past year, have you lost competition or training time due to a shoulder or elbow problem?	☐	☐
4. Do you have any diagnosed shoulder or elbow problem other than a stress or acute injury?	☐	☐

If yes, what? ____

5. Have you received treatment for your shoulder or elbow injury?	☐	☐

If yes, what type of treatment? (You can choose more than one option)

☐ Rest ☐ Rehabilitation ☐ Surgery (what surgery?) ________________

Describe at which level your currently compete at in your sporting event:

Use from the following possible terms: Elite athlete (Olympic athlete / professional athlete / semi-professional athlete), competitive athlete, fitness- or hobby athlete

6. What is the highest level you have competed in? ____
7. At which level do you compete in at the moment? _______________________________________
8. If your current competitive level is not the same as your previous level, is this a direct result of your upper limb injury? ☐ ☐

Choose **ONE** of the following options that best describes your current situation:

☐ I participate fully in sport without upper limb problems ☐ I participate in sport but I have a problem in my upper limb ☐ I do not participate in sport because of my upper limb problem

Instructions to athlete:

The following questions are related to your physical performance in competition and training situations. All questions are related to your **shoulder and elbow**, unless otherwise stated. Mark on the line with an **X** too show the point that best suits your current situation.

1. Do you have problems warming up before a competition or training?

I never feel warmed up before competition or training	Warming up lasts the usual duration

2. How much pain do your experience in your shoulder or elbow?

Pain at rest	No pain during competition

3. How much weakness and/or tiredness (e.g. muscle weakness) do you experience in your shoulder or elbow?

Weakness and tiredness prevent all competitive participation	No weakness, only normal tiredness following participation

4. How unstable does your shoulder or elbow feel during competitive participation?

Regularly sublocates /goes out of place	Completely stable

5. How much has your upper limb problem affected your relationships with coaches, team management and agents?

I left my team or was moved to another team, my contract was terminated or my stipend support was withdrawn	No effect

The following question relate to your competitive performance in your sport. Mark on the line with an **X** too show the point that best suits your current situation.

6. How much have you changed your throwing, bowling or stroke/pull technique etc because of your upper limb injury?

Completely changed, I don't perform that movement anymore | No change in technique

7. How much has your movement speed or movement force (power) suffered/changed due to your upper limb injury?

My movement speed has completely decreased and I have changed sport | No changes in movement speed or power

8. Does your upper limb injury limit your competition/competitive endurance?

Limits completely (I have e.g. changed playing position or changes to a shorter competitive distance/event) | No limit in competitive endurance

9. How much has your upper limb injury reduced/decreased your movement control during e.g. throwing, bowling, stroke etc?

Movement control is unpredictable in throwing, bowling, stroke etc. | No effect on movement control

10. How much do you feel that your upper limb injury has had on your competitive level in your sport (e.g. (for example) does your upper limb (injury) prevent your using your full performance potential)?

I cannot compete and have been forced to change sporting event | I compete at my expected level
